# Relying on Trust: A Qualitative Study on Home‐Dwelling Older Adults' Experiences and Preferences With Multimorbidity Management in Municipal Healthcare Assessments

**DOI:** 10.1111/hex.70445

**Published:** 2025-09-24

**Authors:** Turid R. Aarønes, Kristin Taraldsen, Linda A. H. Kvæl

**Affiliations:** ^1^ Department of Rehabilitation Science and Health Technology OsloMet – Oslo Metropolitan University, Faculty of Health Sciences Oslo Norway; ^2^ Department for Research, Innovation, Education, and Health Service Development Møre og Romsdal Hospital Trust Ålesund Norway; ^3^ Department for Ageing Research and Housing Studies, NOVA – Norwegian Social Research Institute OsloMet – Oslo Metropolitan University Oslo Norway; ^4^ Health Services Research Unit (HØKH) Akershus University Hospital Lørenskog Norway

**Keywords:** multidimensional assessment, multimorbidity, municipal healthcare, Norway, patient participation, person‐centred care, trust

## Abstract

**Background:**

Older adults with multimorbidity present a unique challenge to municipal healthcare due to the complexity of managing multiple health conditions simultaneously. Addressing multimorbidity, especially in older adults, requires an integrated, holistic approach focused on person‐centred healthcare and patient participation. Multidimensional assessments in municipal healthcare, which consider physical, psychological, and social factors, are essential to understanding and managing these complex health conditions. Additionally, trust in the system's reliability and in staff competency is crucial for ensuring quality care.

**Aim:**

The aim of this study was to explore the experiences and preferences of older adults with assessments in the management of multimorbidity within municipal healthcare.

**Methods:**

Qualitative data were obtained through semi‐structured individual interviews with 12 home‐dwelling older adults with multimorbidity from three Norwegian municipalities. The participants were aged 67–92, with an average of five chronic conditions. Interviews were conducted face‐to‐face in participants' homes between February 2023 and February 2024. Interviews were transcribed and analysed using reflexive thematic analysis.

**Results:**

We identified three main themes: nurturing trust and security through professional competence and assessment skills, navigating vulnerability and dependence in healthcare systems, and the impact of patient participation on assessment outcomes. The results emphasise the importance of competence, trust, autonomy, collaboration, and effective communication both in clinical interactions between health professionals and patients and as a system approach in the management of multimorbidity.

**Conclusions:**

Older adults with multimorbidity valued professional competence and effective communication in their healthcare assessments, as these fostered personal and system trust, as well as a sense of security. Participants also highlighted the importance of their involvement in treatment decisions and the need for a coordinated, person‐centred approach to manage their complex health conditions. Our findings underscore the necessity of enhancing healthcare professionals' communication skills, improving information flow, and actively involving patients in assessment and management processes to better support home‐dwelling older adults with multimorbidity in municipal healthcare.

**Patient and/or Public Contribution:**

This study builds on a recent James Lind Alliance process, which highlighted improving communication with frail older adults as a key area in the management of multimorbidity and one highly relevant to our aim. A reference group, including a user representative, a healthcare professional, and a professor, contributed to the development of the interview guide and formulation of the study aim and provided input on the results. Additionally, the reference group discussed the implications of the findings, contributed on strategies for dissemination, and promoted the integration of the perspectives and priorities of older adults with multimorbidity throughout the research process.

**Abbreviations**
HCP: healthcare professional

## 1 Background

Multimorbidity, the presence of two or more chronic diseases in one person, has become a major healthcare concern in recent years, especially for older adults [1, 2, 3]. Globally, the prevalence of multimorbidity increases with age, affecting over 50% of individuals aged over 60 in noninstitutional community settings, including home‐dwelling older adults [4]. This rising prevalence can be attributed in part to people living longer, which means that more individuals are experiencing functional impairments, leading to more individuals facing diverse challenges associated with aging [1, 2]. Multimorbidity can negatively impact an individual's health outcomes, such as their health status, quality of life, and functional abilities [2, 5]. A holistic and integrated approach to care, one which involves treating the whole person and requires collaboration across and within different levels of care, is crucial due to the complexity and interplay of multiple health conditions [1, 2, 6]. However, disjointed services often fail to consistently improve health‐related outcomes, and researchers advocate the use of more coordinated and person‐centred approaches in healthcare [7, 8, 9, 10, 11, 12].

Municipal healthcare, a central component of the Norwegian public healthcare system, offers local health services and manages care for multiple conditions, including those of older adults with multimorbidity [13]. Within municipal healthcare, person‐centred healthcare is essential in shifting from disease‐focused to individual‐focused care [8, 9, 10, 11]. This model fosters active patient participation in decision‐making, which enhances patients' sense of control and health ownership [9]. Municipal services range from self‐employed physicians to home‐based care, rehabilitation, and reablement services, with treatments increasingly provided closer to home, in line with a policy of ageing in place [13]. The 2012 coordination reform transferred greater responsibilities from specialist to municipal healthcare, such as providing 24‐h care and posthospital discharge services, thereby requiring increased cooperation between sectors. However, cross‐sectoral cooperation remains challenging [13, 14].

Multidimensional assessments, which consider physical, psychological, and social factors, are necessary for municipal healthcare managing multimorbidity in older adults [15, 16, 17]. When they provide a holistic health picture, these assessments facilitate the creation of individualised care plans to meet specific needs [16, 17, 18]. The complexity and diversity of health conditions in this population underscore the relevance of such assessments in understanding and managing their care needs. In this study, we understand assessment as a complex, ongoing process employing both structured and unstructured methods [19]. Beyond standardised instruments, then, assessments incorporate clinical skills, observations, and informal conversations. This approach helps healthcare professionals (HCPs) build trust, gain insights into patients' lives, and understand what matters to them. Additionally, assessments can facilitate interdisciplinary collaboration and continuous information flow, thus aligning stakeholders in patient care. When they link different stages of a patient's journey, assessments can enhance coordination across services through improved communication and shared understanding among HCPs [19].

### 1.1 Trust and Distrust in Municipal Healthcare

Both Norwegian health policy and international practices advocate for older adults to live at home as long as possible, with access to quality healthcare services built on trust [12, 20]. This includes predictability and timely help from skilled personnel, which is especially crucial for older adults with multimorbidity who rely heavily on these services [20, 21].

Niklas Luhmann's theory of trust identifies it as fundamental in cooperative relationships, such as those between older adults and HCPs. Luhmann distinguishes between system trust, which is trust in the dependability and proper functioning of social systems, and personal trust, which arises from transparent and straightforward individual interactions [22]. In healthcare, system trust involves confidence in the system's ability to manage information and provide consistent, high‐quality care, while personal trust is built through reliable, competent, and empathetic interactions with HCPs.

Negative experiences can lead to distrust, which hinder cooperation and complicate decision‐making. Trust reduces complexity and encourages participation in healthcare practices [22, 23]. To prevent distrust, HCPs must consistently demonstrate reliability, competence, and empathy [23]. Both system and personal trust are required in managing the healthcare needs of older adults with multimorbidity and foster their confidence to age in place.

### 1.2 Rationale and Aim of the Study

While the challenges of managing care for older adults with multimorbidity are recognised, its full complexity, including holistic care, treatment guidance based on assessments, and understanding patients' experiences and preferences, remains unclear [11, 24]. A recent scoping review identified core competencies needed in multimorbidity management, particularly interprofessional communication and person‐centred communication [6]. Additionally, a meta‐ethnography revealed older adults' desire for improved interprofessional communication, emphasising its role in integrated care [25]. In a systematic review on integrated care values, most studies were focused on expert views, with only one detailing patient perceptions [26]. However, prior research highlighted the importance of patient engagement, care coordination, and service integration in a person‐centred approach [8, 9, 15, 18, 27, 28, 29, 30].

Despite the growing emphasis on person‐centred care and patient participation, research on the role of assessment for older adults with multimorbidity in municipal healthcare is scarce [24]. Descriptions of core competencies in multimorbidity management, especially regarding interprofessional and person‐centred communication, are lacking [6]. There is a need for further research on stakeholder value differences, particularly from the perspective of older adults, as previous studies have primarily been focused on expert views [26].

Enhancing our understanding of older adults' experiences and preferences can help identify gaps in assessment processes, improve HCP‐patient interaction, and develop personalised care. Aligning with personal goals can elevate quality of life and healthcare outcomes. Therefore, the aim of this study was to explore older adults' experiences and preferences with assessments in managing multimorbidity within municipal healthcare in Central Norway.

## 2 Methods

### 2.1 Design

This qualitative study, guided by a pragmatic approach, employed semi‐structured individual interviews to explore the experiences and preferences of 12 older adults with multimorbidity related to assessments. The methodology, in alignment with our exploratory objective, can generate valuable insights by balancing theoretical comprehension with practical solutions to real‐world issues [31].

### 2.2 Setting

This study was conducted in Central Norway within three municipalities of varied sizes: a medium one with around 13,000 inhabitants, and two larger ones close to 32,000 and 67,000, respectively. Selected for their geographic spread within a county and for their size diversity, these municipalities offered an overview of the region's healthcare context. Municipal healthcare services include home help for practical assistance with daily tasks (both personal and instrumental activities of daily living), home nursing (e.g., wound care, medication distribution, and chronic illness follow‐up), day activity services, food delivery, as well as long‐term care in an assisted living facility or a nursing home [32]. Rehabilitation services are offered as short‐term stays at intermediate care institutions or as reablement services delivered in older adults' homes, focusing on enhancing independence in daily activities and reducing the need for long‐term care [33]. Eligibility for services is determined by the municipalities based on a needs assessment, which includes recommendations from the patient's general practitioner. For some services, such as rehabilitation, a referral from the general practitioner is required. Our multifaceted setting including home care, institutional intermediate care, and reablement services offered a comprehensive background for exploring the experiences and preferences of community‐dwelling older individuals living with multimorbidity.

### 2.3 Sample and Recruitment

We strategically selected participants who, due to their experiences and perspectives, could best provide insight and meaningful responses to our research objectives [34]. This meant selecting participants using the following inclusion criteria: age at or above 65 years, home‐dwelling, having two or more chronic conditions, and recipient of municipal healthcare services. HCPs working in the participating municipalities, who were well‐acquainted with the older persons' health and language skills, identified potential participants and provided them with an informational letter. Persons with severe cognitive impairment, advanced dementia, psychiatric conditions, or insufficient proficiency in Norwegian were excluded.

Older adults willing to participate signed an informed consent form. Their contact details were then forwarded from the HCPs to the first author, who scheduled the interviews. We recruited and included 12 older adults whose rich descriptions and heterogeneity provided sufficient information power to address our research aim, which led us to conclude that further interviews were not necessary [35].

### 2.4 Data Collection

We developed an interview guide to accommodate individual interviews allowing us to explore older adults' experiences and preferences without being constrained by previous literature. The reference group, consisting of an HCP, a researcher, and a user representative, contributed by providing input on key questions and topics to include in the guide. Using the guide, we explored older adults' experiences and preferences concerning assessments and their interactions with health services, covering topics such as everyday life, healthcare interactions, experiences in being assessed, personal safeguarding, and barriers to and facilitators of assessments. After the first two interviews, the guide was updated with adjustments to the wording of the questions to ensure more open‐ended inquiries, allowing participants to elaborate more freely (see interview guide in File S1).

Data were collected between February 2023 and February 2024 through 12 individual, face‐to‐face interviews. Conducted by the first author, each interview lasted between 36 and 75 min and was recorded as an MP3 file. All interviews took place in the participants' homes to create a relaxed and safe environment encouraging open, free‐flowing discussions on the research topic [36]. The home setting was chosen deliberately, as it could help participants feel comfortable and provide additional context and insights into their daily lives [37]. Participants were assured they could take breaks as needed.

### 2.5 Data Analysis

The first author transcribed the interviews verbatim and analysed them using the software tool HyperResearch. An experiential‐oriented reflexive thematic analysis following Braun and Clarke [38] was used. Following a review of the transcripts, all authors convened to identify core findings. Subsequently, the first author coded all transcripts, closely aligning with the material and then developing initial themes from these codes. Eight initial themes were identified and refined through iterative data set analysis by the first author and discussions with the third author. Three main themes were then established and discussed among all authors. Upon interconnecting the initial themes, we recognised a central thread of ‘safety’ and thus elements of ‘trust’ and ‘distrust’ in the participants' experiences and preferences enclosed within the main themes. Table 1 demonstrates the process of assigning code labels to quotes and their subsequent categorisation into initial themes.

**Table 1:** From quote to code and initial theme.

Quote	Code label	Initial theme
‘They ask me about my wellbeing. I tell them I'm fine, and if I feel unsafe, I can call. They assure me I can call anytime, even at night, as there's a night shift staff available’ (P1)	Assurance of 24/7 accessibility to home nurse	Reliance on others

## 3 Results

### 3.1 Participants

Twelve older adults with a mean age of 81 years (SD = 8, ranging from 67 to 92 years) were included in the study. The participants had on average, five chronic conditions (from 2 to 9) and received an average of four municipal healthcare services each (ranging from 1 to 7). All participants were dependent on walking aids or wheelchairs for mobility, both indoors and outdoors. Nine lived alone, and three lived with a spouse. Participants' background characteristics are presented in Table 2.

**Table 2:** Background characteristics of study participants (*n* = 12).

Characteristics	*n* (%)
Gender	
Female	8 (67)
Male	4 (33)
Age (years) (mean = 81, SD = 8.0)	
65–69	1 (8)
70–79	5 (42)
80–89	3 (25)
90–95	3 (25)
Education level^a^	
Low	6 (50)
High	6 (50)
Living situation	
Alone	9 (75)
With spouse	3 (25)
Number of chronic conditions	
2	2 (16)
3–5	5 (42)
> 5	5 (42)
Number of healthcare services	
1	1 (8)
2–3	5 (42)
4–5	5 (42)
> 5	1 (8)
Municipality	
A	4 (33)
B	4 (33)
C	4 (33)

^a^ Education level: Low = primary or lower secondary school. High = upper secondary school or college/university.

Participants received a range of municipal health services, including home nursing, personal assistance, medication management, and various rehabilitation approaches. They also received support services like user‐controlled personal assistance—an alternative way of organising health and care services where individuals tailor practical support to their needs—security features such as personal alarms and electronic medication dispensers, and daily visits, as needed. Some accessed daycare activities and food delivery services.

The chronic conditions most frequently identified among the participants, through self‐reporting and verification by HCPs checking medical records were atrial fibrillation, hypertension, osteoarthritis, and stroke. These were followed by a group of less frequent conditions, including diabetes II, incontinence, multiple sclerosis, osteoporosis, cancer, spinal cord stenosis, arthritis, asthma, cardiomyopathy, chronic kidney failure, chronic obstructive pulmonary disease, heart failure, impaired hearing, ischaemic heart disease, neurological diseases, and rheumatoid arthritis. It should be noted that this list of chronic conditions excludes several rare conditions that were present in only a single participant.

### 3.2 Overview and Main Themes

While some participants did not explicitly recognise the term ‘assessment’ they still considered the actions and evaluations performed by HCPs as fundamental aspects of their care management in municipal healthcare, particularly for those living with multimorbidity. The participants prioritised HCPs' kindness and competence, as well as their own involvement in the care process over the specific terminology used.

Our analysis elicited the following three main themes: nurturing trust and security through professional competence and assessment skills, navigating vulnerability and dependence in healthcare systems, and the impact of patient participation on assessment outcomes. Each theme represents an important aspect of the participants' sense of safety and their trust in (or distrust of) their healthcare interactions. Participants demonstrated trust when they felt their needs were met and competence was displayed, while distrust emerged when these elements were lacking. The active involvement of participants was also identified as essential to person‐centred care and further contributed to their sense of trust and safety. The themes identified in our analysis are illustrated in Table 3 below.

**Table 3:** Themes.

Initial themes	Subthemes	Main themes
Importance of basic HCP knowledge	Balancing professional expertise and relational understanding	Nurturing trust and security through professional competence and assessment skills
Establishing mutual understanding		
Reliance on others	Vulnerable individuals relying on offered assistance	Navigating vulnerability and dependence in healthcare systems
Crucial role of information and communication	Challenges navigating a resource‐constrained health system	
Participant perspective on the health system		
Recognition and validation of participant experience	Valuing individual recognition and positive interactions	The impact of patient participation on assessment outcomes
Impact of limited time on participant experience		
Significance of social connections		

#### 3.2.1 Nurturing Trust and Security Through Professional Competence and Assessment Skills

The participants expressed a strong desire for HCPs to be knowledgeable about both their medical conditions and their overall situations. This included being up to date on patient‐specific issues encountered in practice. Several participants viewed HCPs' knowledge and insight into their situations as essential for their feelings of safety and trust. Conversely, a lack of knowledge and practical competence could lead to participants feeling uncomfortable and insecure, while also feeling sorry for HCPs who might not have received sufficient training. As one participant noted:

They simply lack the proficiency to fulfil the duties they're expected to perform […]. They insist they possess the necessary skills, but they don't […]. Consequently, I find myself in the uncomfortable position of having to request them to seek assistance. ‘Call for help’, I have to say, ‘this isn't working’. They persist, often choosing to struggle alone rather than ask for help. I've observed this pattern on several occasions. The constant anticipation of encountering such situations can be rather taxing; it's a challenging aspect of the whole experience.

(P12)

Many participants described that they received home nursing from several different people, which was fine if they were attentive and able to communicate properly. Participants frequently felt more secure with HCPs who were not only proficient at their job but also shared a bit about themselves and allowed patients to get to know them better. This personal touch from HCPs made participants feel more comfortable compared to HCPs who remained impersonal or distant. As one participant noted:

On the initial visit, two individuals [from the reablement team] asked about my husband and other personal matters. It was a truly enjoyable visit, showing how important it is to communicate effectively. They acknowledged that not everyone shares the same perspectives or observations. Instead of imposing their opinions, they were interested in understanding my feelings and experiences.

(P9)

Several participants emphasised how language barriers made communication with the HCP difficult and how this had led to incidents not being assessed or handled properly. One participant noted:

I completely lost bladder control, leading to a distressing situation. The person who came couldn't understand why I was upset, despite my attempts to explain my concerns [that something must be wrong]. Unbeknownst to them, I had a urinary tract infection, which was a contributing factor [to my loss of bladder control…]. So certain issues might be overlooked [without proper communication].

(P8)

Many participants defined competence as the ability of HCPs to build a strong rapport and perform their roles effectively. They described HCPs who were open, friendly, and knowledgeable as competent and trustworthy. Participants felt more at ease and trustful when HCPs demonstrated preparedness and a thorough understanding of their responsibilities. Conversely, participants expressed anxiety and uncertainty when faced with unprepared HCPs. As one participant noted:

I get the feeling that they're not fully aware of the patients' conditions. They seem to only know their assigned tasks for the day. They may not be expected to know everything, but they ask. When I tell them about my situation, they respond with surprise, which is disconcerting.

(P4)

To summarise, the theme highlights how the ability of HCPs to manage participants with multimorbidity is linked to their professional competence and assessment skills, which combines medical knowledge, interpersonal skills, and preparedness. Participants expressed that competence was important for their trust in HCPs and the healthcare system, as well as their sense of safety.

#### 3.2.2 Navigating Vulnerability and Dependence in Healthcare Systems

The participants explained that they relied on others, including family, friends, and healthcare services, to manage their routines at home. While they were grateful for the support, many also felt anxious about becoming a burden to others. While some participants hoped that rehabilitation would improve their function and situation, others felt they had to come to terms with their current levels of function, accepting that life would never be the same again. Regardless of their outlook, many participants shared a common expectation of being able to rely on receiving necessary healthcare services both here and now and when needed. Participants considered predictable encounters with the service, including regular assessments to adjust help based on their needs, vital for their sense of safety. As one participant noted:

It surely matters a lot. As my need for assistance increases and I receive more help, I'm currently quite independent. However, I'd like to believe that if there comes a time when I can't get out of bed due to stiffness in the morning, help will be available.

(P10)

Participants relied on a certain predictability in everyday life, for example, HCPs coming at the scheduled time and delivering services as expected. Conversely, participants felt anxious and insecure and experienced bad days when they did not know whether they were getting the necessary help or because the help was not tailored to their situation. One participant noted:

I need to complete a certain procedure before I can put on my support stockings. But if the HCPs arrive when I'm only halfway through the procedure, they won't wait for me to finish. When this happens, I know it's going to be a difficult day.

(P8)

Accurate information and effective communication were highlighted as key elements in a successful healthcare system. Many participants expressed satisfaction with the clear updates they received on topics such as exercises and rehabilitation, updates from their general practitioner after specialist visits, and explanations for why certain services could not be provided. This level of communication fostered a sense of safety and predictability for participants.

Participants also felt reassured when they knew HCPs were communicating amongst themselves about their situation and treatment plans. Conversely, participants experienced the negative consequences of insufficient information and communication, highlighting the important role of these in conducting high‐quality assessments. Many participants reported feeling burdened by the responsibility of managing their own care transitions, such as arranging transport from rehabilitation to their homes, often without appropriate guidance. Participants attributed inadequate information flow and communication to a perceived lack of coordination within health services. As one participant noted:

My overall impression of the home nursing in my district is that they lack effective communication amongst themselves. If I share a message with one nurse, it often doesn't get passed on. They seem preoccupied with other tasks and even when they answer my call, they appear rushed and then forget to relay my message to the relevant person.

(P11)

Participants conveyed that their experiences of care, particularly at home, were affected by limited time and staff resources. They reported feelings of compromised security and well‐being due to these constraints. As one participant noted:

The home nursing service has far too little time. As a result, I only get the bare minimum of help, not the ideal level of assistance that I truly need. That's clear.

(P11)

The theme highlights the participants' vulnerability and dependence, emphasising their need for predictability, effective communication, and regular assessments as part of navigating the healthcare process.

#### 3.2.3 The Impact of Patient Participation on Assessment Outcomes

Many participants emphasised their need to be seen, heard, and believed by the HCPs they met to feel understood and to influence decisions about their care. Not all participants were asked ‘What matters to you?’ by HCPs during assessments, although all wanted HCPs to know them and their priorities. Participants expressed a desire to decide for themselves what was most important to them, and some felt that decisions made without their involvement suggested that their opinions and needs were not valued. As one participant noted:

I applied for evening supervision, for someone to pop in and say hello, how are you doing…But I haven't received that. They were there for only two evenings and then decided I didn't need it. No, it wasn't justified; they said I had no use for it.

(P7)

Many participants described limited control over their healthcare services, such as scheduling exercise, choosing rehabilitation locations, or selecting home service staff. However, some reported positive experiences from actively collaborating with HCPs on medication, exercise, and rehabilitation stays. This active involvement provided a sense of control and fostered mutual understanding with the providers, which was essential for trust in the HCPs and for receiving personalised treatment based on regular assessments. Participants considered such individualised treatment important for managing their conditions and improving their quality of life. As one participant noted:

I was aware of the treatment options they offered. After my personal assessment with them, we collaborated as a team and decided to initiate a certain treatment plan, with the understanding that we would adjust as necessary.

(P8)

Participants emphasised the importance of a good relationship with HCPs for successful assessments and treatments. Many shared how such relationships facilitated collaboration on decisions about treatments and aids that suited their daily routines. As one participant noted:

She's been here frequently, and we've built a great relationship. We discuss my needs, which she takes very seriously. She's always been mindful of them. Thanks to her, I have all the aid I need.

(P3)

Participants indicated that for a good relationship with HCPs, they needed to find them trustworthy and genuinely interested in their lives. It was also important that HCPs included them in significant care decisions. However, many participants voiced a need for autonomy at home and expressed frustration and feelings of helplessness when HCPs ignored their preferences or behaved rudely in their homes. As one participant noted:

I dislike others' control, especially when I'm giving my all. Yes, she and I lack similar chemistry… My son advises me not to speak ill of her, reminding me of my need for ongoing help. He insists I avoid criticising her, a sentiment I agree with. I plan to keep these thoughts to myself, but I do wonder if she should visit less, given she doesn't seem to like me much, either.

(P3)

This theme highlights the importance of participants having input during assessments and in their treatment decisions, actively collaborating with HCPs, and maintaining autonomy despite their reliance on health services.

## 4 Discussion

We identified three key themes from older adults' experiences and preferences with multimorbidity assessments in the context of municipal healthcare. First, HCPs' professional competence and assessment skills, including medical knowledge, interpersonal skills, and preparedness, were required to foster safety and personal trust. Second, system trust in receiving necessary assistance and timely care was based on participants' reliance on others in their daily lives and on the provision of predictability, effective communication, and regular assessments. Third, acceptance of their input during assessments and in treatment decisions, and actively collaborating with HCPs, was highly valued. These themes capture the conditions for optimal care in municipal healthcare settings.

Participants underlined the importance of HCPs' medical know‐how, interpersonal skills, and preparedness, associating these with feelings of personal trust and safety during interactions and assessments. This supports existing research stressing the need for a balance between performance and relational skills in proficient HCPs [39]. Performance skills, including theoretical knowledge, technical expertise, decision‐making capabilities, and self‐awareness of limitations, are fundamental to precise assessments, successful interventions, personal trust, and cooperation [39, 40]. Reinforcing previous research [40], participants in our study expressed more personal trust in HCPs who demonstrated knowledge, answered their queries, and proficiently used disease management equipment. Conversely, a lack of practical knowledge and skills incited stress, anxiety, and doubts about care quality, indicating distrust.

Our findings suggest that fostering personal trust goes beyond just skills; it requires HCPs to be familiar with older adults' medical history before interactions. In line with existing research [39, 40], participants linked competent HCPs with open communication, empathy, and relationship‐building and valued those who cultivated personal trust through effective and culturally sensitive communication [6]. However, language barriers, poor inter‐HCP communication, and unpreparedness often resulted in overlooked care issues and perceived inferior care quality. These findings align with those of studies emphasising communication in high‐quality care for individuals with multimorbidity [6, 25, 29]. Training HCPs in communication, for example through simulation or real‐life scenarios, could thus improve patient‐centred care by enhancing their skills in addressing older adults' needs and fostering personal trust [6]. Our results indicate that HCPs' competence and assessment skills can build personal trust in older adults with multimorbidity, a vulnerable group with limited control over their healthcare encounters. According to Luhmann, personal trust simplifies complexity, becoming key in healthcare and particularly in managing the complex health needs of older adults with multimorbidity [22, 23]. Thus, participants' personal trust in HCPs was largely based on their perceived competence and responsiveness to the participants' needs.

The participants in our study underscored the importance of understanding the perspectives of older adults with multimorbidity, which highlighted their vulnerable position and dependence on others for daily care. This reliance extends beyond interpersonal relationships with HCPs to encompass system trust in the broader healthcare system. For these individuals, system trust is crucial, particularly in the context of ageing in place policies which aim to help older adults live independently at home for as long as possible [12].

Participants emphasised that their trust in the healthcare system was contingent upon the system's ability to provide consistent, coordinated, and high‐quality care at home. In line with previous research, this involves not only the competence and assessment skills of individual HCPs but also the effectiveness of organisational factors such as efficient communication protocols, person‐centred policies, and adequate resources and support for HCPs delivering home‐based services [8, 18, 29].

Luhmann's theory suggests that trust in the healthcare system is essential for older adults with multimorbidity to effectively manage their complex health needs [22]. System trust simplifies the intricacies of healthcare and fosters a sense of security and control over older adults' health management when receiving care in their own homes. Our participants needed to trust that their values would be respected and that they would receive necessary help in a timely manner, which ultimately enhances health outcomes [23].

However, our findings suggest some participants were not always asked about their main concerns during HCP assessments or other interactions, which indicates a gap in effective communication and patient engagement. A trusting environment where information is freely shared is required to develop care plans that align with the values and needs of older adults. Regular assessments that consider patient values can foster trust and improve health outcomes. These findings are in line with Health Education England's framework which advocates for open HCP‐patient communication and valuing individual perspectives in shaping personalised care plans [41]. For instance, respecting routine preferences can enhance an individual's comfort and dignity, while HCPs with strong communication skills can conduct person‐centred assessments that identify and respond to the individual's main concerns [6]. While some participants expressed satisfaction with their care, others reported unmet needs, which underscores the importance of HCPs having adequate time and resources to build relationships and meet individuals' needs [6, 28, 29].

Our findings reflect participants' desire for involvement in assessments and treatment decisions, as well as positive collaboration experiences. Patient participation can integrate older adults and their relatives in decision‐making [42], and is a key aspect of person‐centred healthcare [8, 9, 29]. It counters the ‘doctor knows best’ attitude, signalling a shift towards person‐centred care [43]. However, focusing solely on patient autonomy can overlook necessary medical expertise, which can potentially cause patient anxiety and inconsistent health outcomes. Thus, a holistic approach incorporating both patient and medical expertise is needed. In our study, participant involvement and HCP collaboration entailed being acknowledged and co‐creating care decisions. Lack of patient input during assessments and overlooked needs led to participant insecurity and frustration. These findings align with past research showing that understanding how care and communication priorities can differ between older individuals with multimorbidity and HCPs is key for tailored interventions [27, 29].

Participants in our study relied heavily on family and friends for support, which often provided safety but could also feel burdensome, in line with previous research [29, 30]. Our participants' reliance could trigger feelings of lost autonomy, insecurity, and anxiety. Active involvement during assessments and care can restore a sense of control and lessen anxiety. Open communication and collaboration build personal trust, which is central for older adults with multimorbidity to feel secure in their care; thus, they enhance satisfaction and health outcomes [6, 8, 29, 30, 41]. These findings align with Luhmann and highlight that personal trust and distrust manage social complexity in patient participation [22]. Personal trust can promote active participation and simplify health complexity, while distrust may inhibit engagement and create communication barriers [23]. Hence, fostering personal trust and addressing distrust are needed to encourage patient participation.

### 4.1 Implications for Practice and Research

The findings of our study underline the necessity for HCPs to demonstrate professional competence, assessment skills, and preparedness when interacting with older adults in managing multimorbidity. These qualities build trust between older adults with multimorbidity and the healthcare system. Personal and system trust are particularly important for this population due to their frequent and complex interactions with various healthcare services, which require coordinated and consistent care.

The study highlights the significant role of comprehensive assessments, which are an integrated part of HCPs' collaborations with older adults, conducted within a framework of person‐centred care. These assessments are important for building and sustaining personal trust. Effective communication and personalised care strategies help ensure that the unique needs and preferences of older adults with multimorbidity are met, which reinforce their trust in the healthcare system. Additionally, a systems perspective, ensuring that healthcare policies and organisational structures effectively support these priorities is essential. Ongoing research should explore methods to integrate these elements into the management of multimorbidity in municipal healthcare to ensure a coordinated, efficient approach.

### 4.2 Strengths and Limitations

The study's qualitative approach provides a rich understanding of older adults' experiences and preferences with the management of multimorbidity in municipal healthcare in Central Norway. While the limited number of participants may pose representational challenges, the diversity in age, sex, health conditions, and living situations within our sample contributes to a broader understanding of older adults' experiences and preferences across different settings. To mitigate potential pressure on older adults from HCPs to take part in the study the first author assured them that participating was voluntary and that they could withdraw at any time without consequences. To counter the inherent challenges of reliance on self‐reported data and possible reflexivity issues, a warm and relaxed atmosphere was cultivated during the interviews. Participants shared their experiences freely, and these encompassed both positive and negative aspects. In addition, the study's credibility was enhanced by involving relevant stakeholders in our reference group, and its trustworthiness was reinforced by the authors' combined clinical and research expertise in healthcare services for older adults. Findings are presented in compliance with the Standards for Reporting Qualitative Research (see SRQR checklist in File S2).

With broad practical and research experience in healthcare services for older adults, the authors' knowledge added depth to the data interpretation and helped identify any preconceived notions. The authors hope that this combination of expertise and awareness contributed to the credibility and trustworthiness of the study's results.

## 5 Conclusion

In this study, we found that older adults with multimorbidity valued professional competence and effective communication in their interactions with HCPs, as these fostered personal and system trust and a sense of security. Our participants emphasised the importance of being involved in treatment decisions and the need for a coordinated, person‐centred approach to manage their complex health conditions. To improve their experiences and outcomes, it is suggested to strengthen HCPs' medical and interpersonal skills, streamline provider‐patient communication, and incorporate patients' perspectives. Co‐designing services with home‐dwelling older adults and their caregivers could help municipal healthcare systems better address the complex needs of individuals with multimorbidity. Future research should explore how to operationalise and scale such codesign initiatives in municipal healthcare settings.

## Author Contributions

**Turid R. Aarønes:** conceptualisation (lead), writing – original draft (lead), formal analysis (lead), writing – review and editing (lead). **Linda A. H. Kvæl:** conceptualisation (supporting), formal analysis (supporting), writing – review and editing (equal). **Kristin Taraldsen:** conceptualisation (supporting), writing – review and editing (equal).

## Ethics Statement

The study underwent review from Regional Committees for Medical and Healthcare Research Ethics (REK; No. 533642) and was processed by the Norwegian Agency for Shared Services in Education and Research (Sikt), ensuring it met all necessary privacy regulations for personal data management (No. 544851). All data were securely stored on the Services for Sensitive Data (SSD) platform, in accordance with Norwegian privacy laws. To protect the confidentiality of informants, the identities of municipalities were anonymised. Participants were provided with both written and verbal information about the study and personal data handling, and they gave their written informed consent before their involvement. They were made aware of their right to participate voluntarily, with the freedom to withdraw at any time without repercussions. On the day of their individual interviews, each participant was briefed about the objectives, the use of audio recordings, the methods of anonymisation, and the option to take breaks as necessary.

## Conflicts of Interest

The authors declare no conflicts of interest.

## Supporting information

Additional file 1.

Additional file 2.

## Data Availability

As the data collection approval for this study states the data to be available only to the researchers, data and materials collected for this manuscript will not be shared.
