# Supplementary material for: Relying on Trust: A Qualitative Study on Home‐Dwelling Older Adults' Experiences and Preferences With Multimorbidity Management in Municipal Healthcare Assessments
Source: Health Expect. 2025 Sep 24;28(5):e70445. doi: 10.1111/hex.70445 (PMC12457978; doi:10.1111/hex.70445)
Supplement: Supplementary file 1 — Additional file 1. [file HEX-28-e70445-s002.docx]

Additional file 1

# **Interview guide – Individual interview**

| **ENTRY QUESTIONS** |
| --- |
| 1. Could you please describe your day-to-day life to me?  - You have had interactions with the health service; could you please share your experiences? - Could you please tell me about the assistance you receive from the home care service? - Could you please provide your experiences related to these situations? |
| **ASSESSMENT METHODS AND EXPERIENCES** |
| 1. Could you please discuss instances where you have been assessed or examined by healthcare personnel?  - Could you kindly provide a specific example? - How did you feel about being examined? - What are your lasting impressions from these experiences? |
| **SAFEGUARDING** |
| 1. When you were assessed, how did you perceive you were treated as an individual?  - Could you please share what you think the purpose of the assessment was? - How do you view the health service and their focus? - Could you please describe the outcomes of the assessment? - How was the assessment followed up? What are your thoughts on the follow-up? |
| **BARRIERS AND FACILITATORS FOR ASSESSMENT** |
| 1. Could you please detail what went well and what did not during your assessment or examination?  - In what way did the assessment capture what mattered to you? - Could you describe what you consider important during assessment? - How did the healthcare personnel identify or miss what was important to you? - Could you please share your thoughts on the conditions of the assessment, such as the duration and environment? - Could you please share your impressions of the healthcare personnel who conducted your assessment? - Are there any aspects we might be missing with the current tools or methods? If so, could you please share what those might be?  1. If you were to be a patient for the first time again, could you please describe what an ideal assessment situation or interaction would look like for you? |
| **CONCLUDING QUESTION** |
| 1. Are there any other significant points about your experiences and preferences with assessments that you would like to share or discuss? |
